# Bisphenol A exposure promotes HTR-8/SVneo cell migration and impairs mouse placentation involving upregulation of integrin-β1 and MMP-9 and stimulation of MAPK and PI3K signaling pathways

**DOI:** 10.18632/oncotarget.17882

**Published:** 2017-05-16

**Authors:** Xi Lan, Li-Juan Fu, Jun Zhang, Xue-Qing Liu, Hui-Jie Zhang, Xue Zhang, Ming-Fu Ma, Xue-Mei Chen, Jun-Lin He, Lian-Bing Li, Ying-Xiong Wang, Yu-Bin Ding

**Affiliations:** ^1^ Department of Reproductive Biology, School of Public Health, Chongqing Medical University, Chongqing, 400016, P.R. China; ^2^ Department of Immunology, School of Medicine, University of Pittsburgh, Pittsburgh, Pennsylvania, 15260, USA; ^3^ Center of Molecular Diagnostic Medicine, Life Science Institute, Chongqing Medical University, Chongqing, 400016, P.R. China; ^4^ Ministry of Education Key Laboratory of Diagnostic Medicine, College of Laboratory Medicine, Chongqing Medical University, Chongqing, 400016, P.R. China; ^5^ The Key Laboratory of Birth Defects and Reproductive Health of the National Health and Family Planning Commission, Chongqing Population and Family Planning Science and Technology Research Institute, Chongqing, 401147, P.R. China

**Keywords:** bisphenol A, extravillous trophoblasts, cell migration, placentation, MAPK/PI3K signaling pathway

## Abstract

In this study, we investigated the effect of Bisphenol A (BPA), an endocrine-disrupting chemical, on the migration of human trophoblasts and mouse placentation by using the primary extravillous trophoblast (EVT) and its cell line HTR-8/SVneo, villous explant cultures, and pregnant mice. BPA increased EVT motility and the outgrowth of villous explants in a dose-dependent manner. BPA also increased the protein levels of integrin-β1 and matrix metalloproteinase (MMP)-9 in human EVTs. Low-dose BPA (≤50 mg) increased the protein levels of MMP-9 and MMP-2 as well as integrin-β1 and integrin-α5 in mouse placenta and decreased the proportion of the labyrinth and spongiotrophoblast layers. Inhibitors of mitogen-activated protein kinase (MAPK) U0126 and phosphatidylinositol-3-kinases (PI3K) LY294002 reversed the protein levels of integrin-β1 and MMP-9 as well as the migratory ability induced by BPA. In conclusion, these results indicated that BPA can enhance trophoblast migration and impair placentation in mice by a mechanism involving upregulation of integrin(s) and MMP(s) as well as the stimulation of MAPK and PI3K/Akt (protein kinase B) signaling pathways.

## INTRODUCTION

Bisphenol A (BPA) is an endocrine-disrupting chemical that is used extensively in the production of polycarbonate plastics and epoxy resins. The general population, including women of reproductive age and pregnant women, is exposed to BPA in daily life [1]. Contamination by this chemical can occur, for example, through the consumer's skin or by ingestion of personal care products [2]. Adverse effects of BPA on reproductive outcomes and early development are possible [3]. It has been suggested that exposure to BPA may be a detrimental factor related to a decline in female fertility [4]. Oogenesis impairment, reduced embryo implantation sites, and even sociosexual behavioral changes in mice and rats were observed after BPA exposure [5–8]. BPA also leads to compromised *in vitro* decidualization in human endometrial stromal cells [9].

BPA may interfere with the biological function of trophoblast and placenta [10]. *In vitro* studies show that BPA exposure severely disrupts the expression of human placental genes and of several prominent placental hormones/factors in trophoblast cells isolated from human placentas at term [11]. Trophoblast cell invasion, proliferation, apoptosis and differentiation were significantly altered upon BPA exposure [12–16]. Moreover, the rapid movement of BPA was observed across the term placenta and BeWo cell line monolayer [17]. Mediated by P-glycoprotein and the ATP-binding cassette sub-family G member 2 (ABCG2) transporter proteins, BPA may stimulate drug efflux in human placenta [18, 19]. In mouse and rat *in vivo* studies, BPA disrupts placental development and leads to degenerative changes, which lead to reproductive disorders [20–23].

The migration of trophoblast is of profound significance during embryo implantation and early embryonic development [24–27]. Excessive trophoblast invasion is involved with placenta accreta, increta, percreta or choriocarcinoma [28]. In this study, we aimed to characterize the effects of BPA on trophoblast motility and placentation as well as their possible mechanisms, which may present insights into reproductive toxicity of BPA.

## RESULTS

### HTR-8/SVneo cell proliferation was not changed by BPA treatment

**Figure 1:**
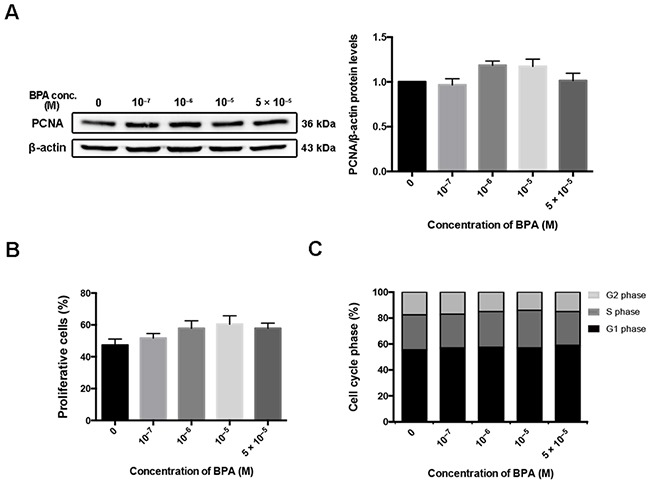
HTR-8/SVneo cell proliferation after BPA treatment **(A)** Western blot detection of PCNA in HTR-8/SVneo cells treated with different doses of BPA for 48 h. β-actin was used as the loading control. The bands were quantified, normalized by β-actin, and presented relative to control (1.0) as means ± SEM. **(B)** Proliferative cells detection by EdU assay. The proliferation rate was quantified by counting the number of EdU-positive cells in each of five randomly selected fields. **(C)** Flow cytometry evaluation of cell cycle and the percentage of cells in G1, G2, and S phase.

The effect of BPA on the viability of the EVT cell line HTR-8/SVneo was determined by flow cytometry (Supplementary Figure 1). No significant difference in cytotoxicity was observed when cells were treated with 10^–7^–5 × 10^–5^ M BPA. The protein level of proliferating cell nuclear antigen (PCNA), a cell proliferation marker, did not change after BPA treatment (Figure 1A). In addition, flow cytometry and 5-ethynyl-2'-deoxyutidine (EdU) assay confirmed that exposure to the above concentrations of BPA had no significant impact on cell proliferation of the HTR-8/SVneo cell line (Figure 1B, 1C; Supplementary Figure 2).

### BPA increased the motility of HTR-8/SVneo cells

**Figure 2:**
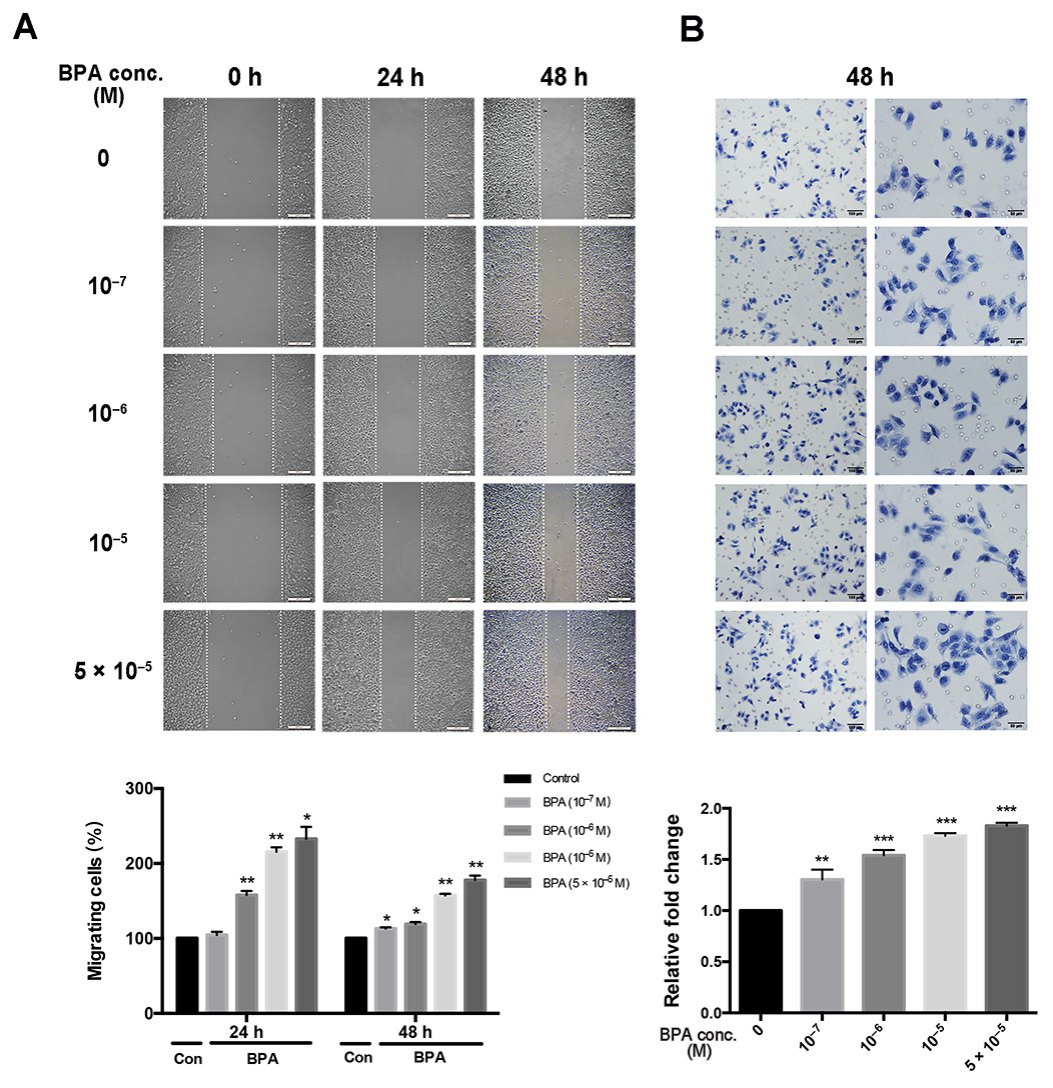
Migration of HTR-8/SVneo cells treated with different concentrations of BPA Scratch and Transwell assays were performed after 48 h treatment of BPA. **(A)** Migration of cells by scratch assay. Significant differences between BPA-treated and control groups are indicated (*P < 0.05, **P < 0.01). Scale bar, 200 μm. **(B)** Migration of cells by Transwell assay. **P < 0.01, ***P < 0.001. Scale bar, 100 μm or 50 μm.

A monolayer scratch assay was performed to assess the ability of HTR-8/SVneo cells to migrate away from a confluent monolayer. The distance (in micrometers) traveled by the cells to populate the cell-free gap created by the scratch wound was calculated and plotted. Higher concentrations of BPA increased the migratory ability of the cells toward the scratched area compared to that obtained with untreated controls within 24 h and 48 h of scratching (Figure 2A). Similar to the results of the wound assay, the migratory ability of HTR-8/SVneo cells, assessed by the Transwell assay, was also altered in response to BPA (Figure 2B).

### BPA induced the migration and outgrowth of villous explants

**Figure 3:**
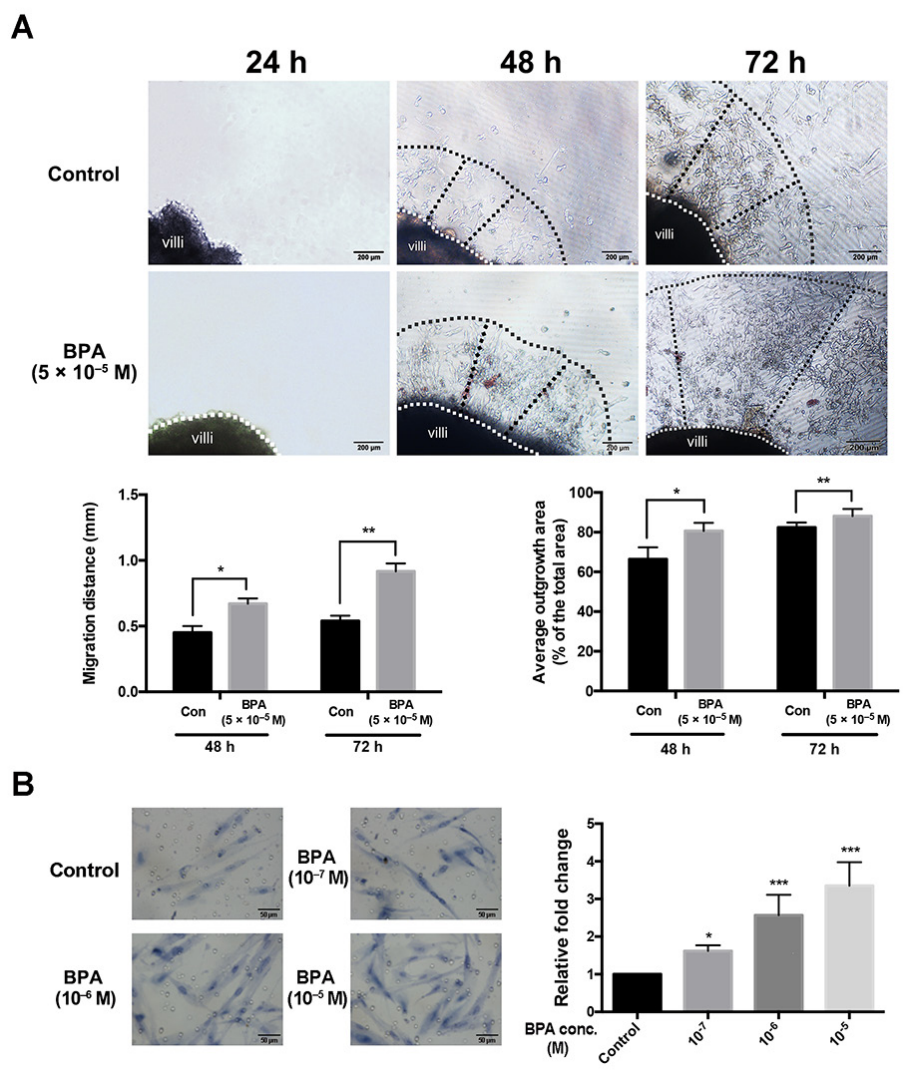
Migration and outgrowth of the trophoblast from explanted first trimester placental villi in the presence and absence of BPA **(A)** Villi were treated with BPA for 72 h. Dashed lines represent the column base (white) and invasive extremity (black). The Matrigel explant assay was performed in duplicate. *P < 0.05, **P < 0.01. Scale bar, 200 μm. **(B)** Transwell migration assay detection the effect of BPA on primary EVTs migration. The primary EVTs were treated with BPA at the concentrations of 10^-7^ M, 10^-6^ and 10^-5^ M. *P < 0.05, *** *P* < 0.001. Scale bar, 50 μm.

To further confirm the impact of BPA on trophoblast migration *in vivo*, each explant was obtained from a single placenta and separated into two groups which were treated with BPA (5 × 10^–5^ M) or vehicle control. Bright field images showed that there were significantly more trophoblast cells in the outgrowth area from villous tip in BPA-treated explant cultures compared to that observed in the controls. The average outgrowth lengths were 0.54 mm and 0.91 mm in explants treated for 48 h and 72 h with BPA, respectively; while the average outgrowth lengths were 0.45 mm and 0.67 mm at 48 h and 72 h, respectively, in the control explants (Figure 3A). The ratio of the outgrowth area compared to the total area of the explant in BPA-treated group was 82.3% and 87.5% at 48 h and 72 h, respectively; the ratio was 67.0% and 80.6% in the control at 48 h and 72 h, respectively (Figure 3A). Similarly, the results showed a dose-dependent increase in migration of primary EVT cells after exposure to BPA (Figure 3B).

### Protein level of MMP-9 after BPA exposure and the effect of MMP-9 on cell migration

**Figure 4:**
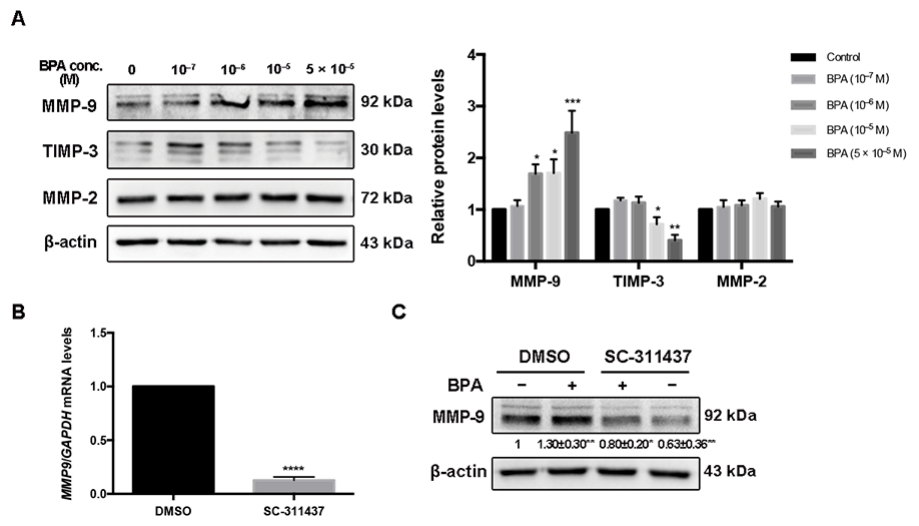
MMP-9, TIMP-3 and MMP-2 expression in HTR-8/SVneo cells exposed to BPA **(A)** Western blot analysis of protein levels of MMP-9, TIMP-3 and MMP-2. *P < 0.05, **P < 0.01, ***P < 0.001. **(B)** RT-qPCR detection of mRNA level of MMP-9 after inhibition by 5 μM SC-311437 for 24 h. ****P < 0.0001. **(C)** Protein level of MMP-9 after treatment with BPA (10^-6^ M) together with or without SC-311437. *P < 0.05, **P < 0.01.

**Figure 5:**
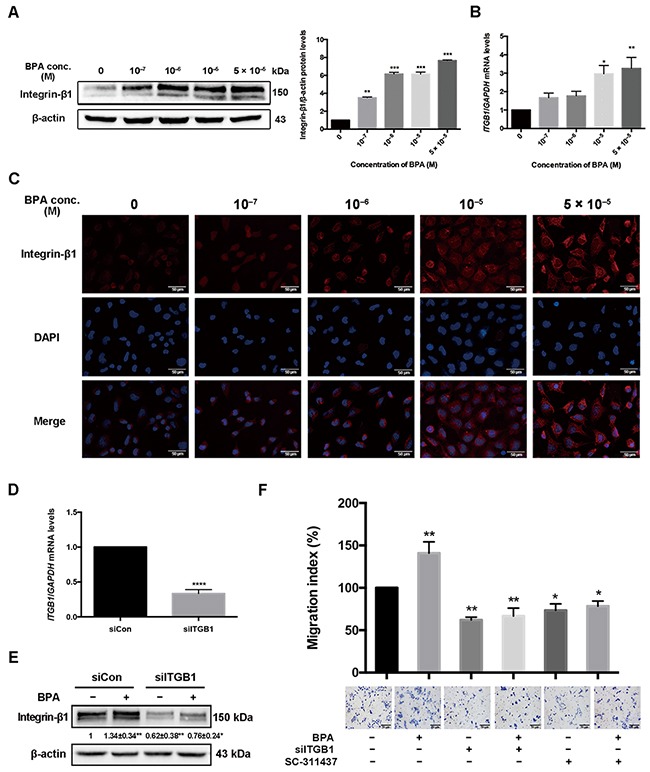
Integrin-β1 (*ITGB1*) expression in HTR-8/SVneo cells treated with BPA **(A, B)** *ITGB1* protein and mRNA levels in HTR-8/SVneo cells treated with BPA were determined by western blot (A) and RT-qPCR (B). *GAPDH* was used as control for RT-qPCR and β-actin for western blot. *P < 0.05, **P < 0.01, ***P < 0.001. **(C)** Immunofluorescence staining of integrin-β1 in HTR-8/SVneo cells treated with BPA. Blue indicates DAPI staining, whereas red (Cy5) indicates integrin-β1. Scale bar, 50 μm. **(D)** RT-qPCR detection of mRNA level of integrin-β1 in cells transfected by 2 μM siRNA for 48 h. ****P < 0.0001. **(E)** Protein level of integrin-β1 after treatment with BPA (10^-6^ M) with or without siRNA transfection. *P < 0.05, **P < 0.01. **(F)** Transwell assay detection of HTR-8/SVneo cell migration co-treated with si-ITGB1 and MMP-9 inhibitor SC-311437. *P < 0.05, **P < 0.01. Scale bar, 100 μm.

BPA (5 × 10^–5^ M) increased the protein level of MMP-9 to 246.3% of that of the control; however, the level of matrix metalloproteinase (MMP)-2 did not change. In addition, the protein level of TIMP-3 decreased in a dose-dependent manner following BPA exposure (Figure 4A). The protein levels of both MMP-9 and MMP-2 increased after BPA treatment of primary EVTs (Supplementary Figure 3A). The effects of MMP-9 inhibitor SC-311437 on cell viability and *MMP-9* expression were assessed prior to the migration assay (Figure 4B, 4C, Supplementary Figure 4). The protein level of MMP-9 in cells co-treated with BPA and SC-311437 was weaker than that treated with BPA alone (Figure 4C). The number of cells that migrated across the Transwell insert membrane in BPA- and SC-311437-treated cells was reduced by 55.65% of that of the BPA-treated cells (Figure 5F).

### Integrin-β1 level after BPA exposure and the effect of integrin-β1 on cell migration

The mRNA and protein levels of integrin-β1, an extracellular matrix (ECM) transmembrane receptor, increased in HTR-8/SVneo cells upon BPA treatment (Figure 5A, 5B). Stronger integrin-β1 intensity was shown in BPA exposed cells than those in the control (Figure 5C). However, the level of integrin-α5, another ECM transmembrane receptor, was not altered in HTR-8/SVneo cells after BPA exposure (Supplementary Figure 5). The upregulation of integrin-β1 and integrin-α5 was also observed in primary EVT cells (Supplementary Figure 3B). si-ITGB1 knockdown efficiency was verified by western blot and RT-qPCR (Figure 5D, 5E). It showed that both cell migration and the protein level of integrin-β1 in cells co-treated with BPA and si-ITGB1 were significantly reduced compared to those treated with BPA alone (Figure 5E, 5F).

### BPA treatment did not change mesenchymal and epithelial biomarker levels

**Figure 6:**
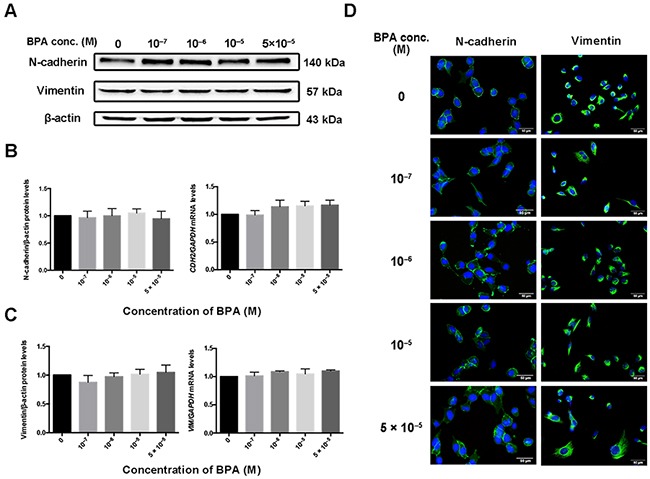
Expression of *CDH2* and *VIM* in HTR-8/SVneo cells exposed to BPA **(A-C)** Protein (A, B) and mRNA (C) levels of N-cadherin (encoded by *CDH2*) and vimentin (encoded by *VIM*) in HTR-8/SVneo cells treated with BPA as determined by western blot and RT-qPCR, respectively. **(D)** Immunofluorescence intensity of N-cadherin and vimentin in cells treated with different concentrations of BPA. Blue indicates DAPI staining, whereas green (FITC) indicates N-cadherin and vimentin. Scale bar, 50 μm.

BPA exposure did not change the protein and mRNA levels of mesenchymal markers vimentin or N-cadherin (encoded by *CDH2*) in the HTR-8/SVneo cell line (Figure 6A–6C). Immunofluorescence staining confirmed these results (Figure 6D). Similarly, the protein level of N-cadherin and epithelial marker E-cadherin did not change in primary EVTs exposed to BPA (Supplementary Figure 3C). Since the HTR-8/SVneo cell line dose not express E-cadherin and primary EVTs do not produce vimentin, we did not detect changes in their expression after exposure to BPA.

### MAPK and PI3K signaling pathways participated in BPA-stimulated levels of integrin-β1 and MMP-9 and cell migration

**Figure 7:**
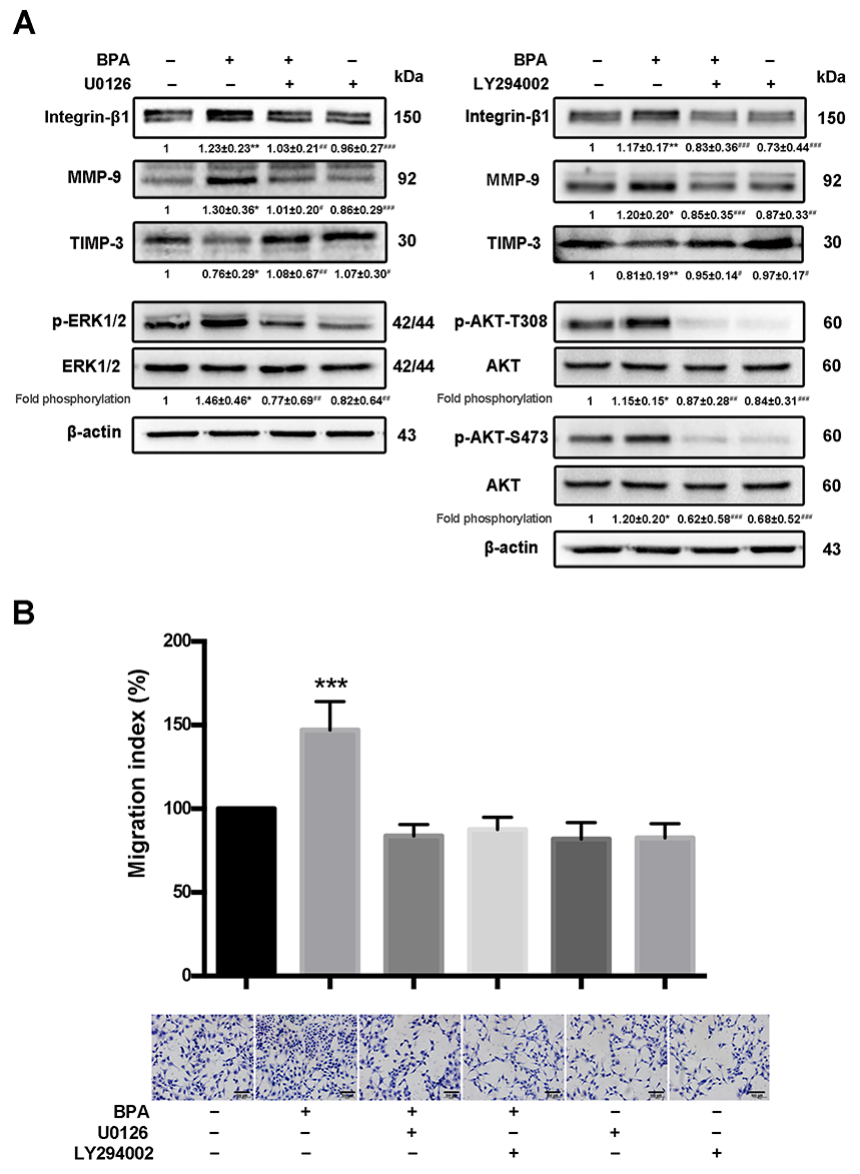
BPA-stimulated HTR-8/SVneo cell migration, MMP-9 and integrin-β1 upregulation, as well as MAPK and PI3K signaling pathways activation **(A)** Western blot analyses of integrin-β1, MMP-9, and TIMP-3 protein levels and phosphorylation levels of ERK and Akt (p-Akt T308 and p-Akt S473) in HTR-8/SVneo cells exposed to BPA for 48 h with or without addition of U0126 (10 μM) and LY294002 (5 μM) for 24 h. *P < 0.05, **P < 0.01, ***P < 0.001. *P < 0.05 compared with vehicle control group; ^#^*P* < 0.05 compared with the BPA treatment group. **(B)** Transwell assay detection of the migration of HTR-8/SVneo cells treated with BPA (10^−6^ M) and/or U0126 or LY294002. The Transwell assay was performed for six times. ***P < 0.001. Scale bar, 100 μm.

The phosphorylation of protein kinase B (Akt, Thr308 and Ser73) and ERK1/2 increased, integrin-β1 and MMP-9 were upregulated, and TIMP-3 was downregulated in BPA-treated HTR-8/SVneo cells compared to those in control cells (Figure 7A). In addition, treatment with the extracellular signal-regulated kinase (ERK1/2) inhibitor U0126 or the PI3K inhibitor LY294002 for 24 h abolished the BPA-induced phosphorylation of ERK and Akt, respectively (Figure 7A). The migration of HTR-8/SVneo cells incubated with BPA in the presence or absence of U0126 or LY294002 were also altered accordingly (Figure 7B).

### MAPK and PI3K signaling pathways were involved in the upregulation of BPA-induced integrin-β1 and integrin-α5 in mouse placentas and impaired placentation

**Figure 8:**
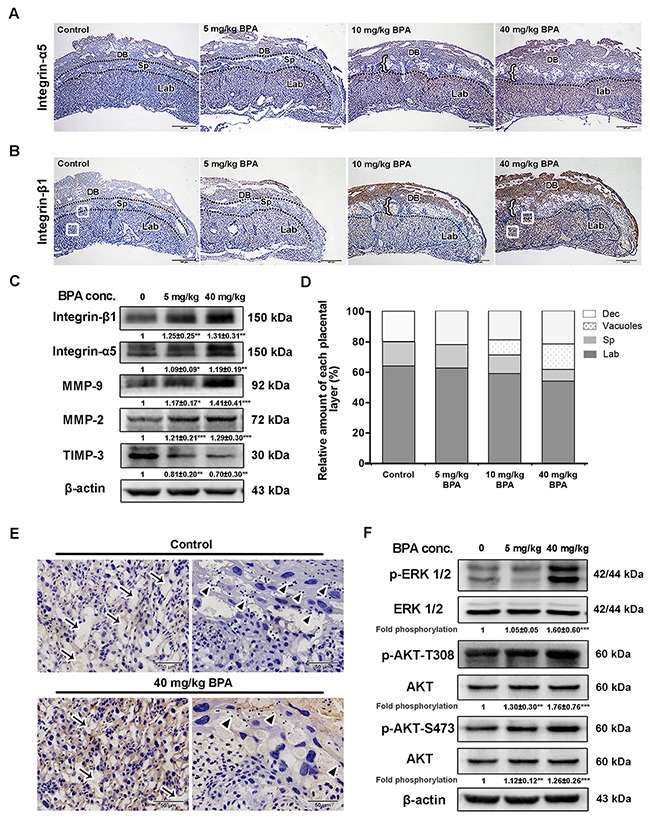
BPA-induced impaired placentation, upregulation of integrins and MMPs as well as activation of MAPK and PI3K signaling pathways in mice **(A, B)** Immunohistochemical detection of integrin-α5 (A) and integrin-β1 (B) in mouse placentas on E14.5. Mice were exposed to BPA from E0.5 to E5.5 of pregnancy. Black dotted lines indicate the boundaries of the placental layers. Brown staining indicates positive staining. DB, decidua basalis; Sp, spongiotrophoblast layer; Lab, labyrinthine layer. Left brace indicates vacuoles obtained from placentas of mice treated with 10 mg/kg and 40 mg/kg BPA. White boxes in the labyrinthine layer in B (control and 40 mg/kg BPA) are enlarged in E. Scale bar, 500 μm. **(C)** Western blot analysis of integrin-β1, integrin-α5, MMP-9, MMP-2, and TIMP-3 in the placenta on E14.5 from mice treated with BPA as in A and B. Values are mean ± SE. *P < 0.05, **P < 0.01, ***P < 0.001. **(D)** Changes in the relative amount of each layer in the E14.5 placenta from mice treated with BPA. **(E)** BPA-induced morphological changing areas enlarged from Figure 8B. Arrows show the intervillous spaces in the labyrinthine layers in mouse placenta. Arrowheads indicate the glycogensome lining within the trophoblast giant cells. Scale bar, 50 μm. **(F)** Analysis of ERK and Akt (T308 and S473) phosphorylation in placentas from mice treated with BPA. Values are mean ± SE. **P < 0.01, ***P < 0.001.

To avoid any adverse effect of high dose of BPA on blastocyst development and implantation, pregnant mice were administrated with low doses of BPA (5 mg/kg, 10 mg/kg, and 40 mg/kg) from E0.5 to E5.5 of pregnancy. The protein levels of integrin-β1 and integrin-α5 were remarkably increased in BPA-treated mouse placentas, especially in the labyrinthine layer and the decidua basalis as detected by immunohistochemistry, whereas they were almost undetectable in control mouse placentas (Figure 8A, 8B). Neither integrin-β1 nor integrin-α5 was expressed in the spongiotrophoblast layer of placentas of the two groups. BPA also enhanced the protein levels of MMP-9 and MMP-2 in mouse placentas and inhibited the level of TIMP-3 (Figure 8C). Meanwhile, the levels of phosphorylated Akt (Thr308 and Ser473) and ERK also increased after 5 days of treatment with 5 mg/kg or 40 mg/kg BPA (Figure 8F).

The relative proportion of placental labyrinth was lower in mice treated with BPA than that of the controls by E14.5 (Figure 8A, 8B, 8D). The spongiotrophoblastic layer was also reduced in mouse placentas treated with the 10 mg/kg and 40 mg/kg BPA compared to that of the controls and the placentas treated with lower dose of BPA (5 mg/kg). Furthermore, the intervillous spaces in the labyrinth were narrowed and reduced in BPA-treated mice than in the control (Figure 8E, arrows). Glycogenosomes were more frequently noted in the control than in the BPA-treated mice (Figure 8E, arrowheads). Notably, large vacuoles were present in the placentas of mice treated with 10 mg/kg and 40 mg/kg BPA (Figure 8A, 8B).

## DISCUSSION

BPA may impair EVT cell proliferation, migration, and invasion, key processes for placentation and pregnancy in mammals [16]. We found that BPA-enhanced migration of EVT in a dose-dependent manner. BPA exposure also stimulates migration of several cancer cell lines *in vitro* [33–37]. However, these results are inconsistent with the finding that BPA inhibits the migratory and invasive abilities of HTR-8/SVneo cells at concentrations of 10^–11^ M to 10^–7^ M [16]. We speculate that cell culture conditions, such as medium composition, pre-treatment of Transwell chamber with collagen and the recovery time after BPA removal, may interfere with cell migration in these studies.

MMP-9, an autocrine factor that localizes primarily to EVT cells at 6–8 weeks of human gestation, regulates trophoblast invasion and migration by degrading ECM [28, 38, 39]. BPA exposure induces expression of *MMP*s and cell migration in several cancer cell types [33, 37]. We confirmed that MMP-9 and MMP-2 were involved in BPA-induced EVTs migration. Previous studies have shown that MMP-9 is the effector molecule of ECM adhesion receptor integrin-β1 [40, 41]. Our study revealed that BPA exposure increased the level of integrin-β1 and integrin-α5 in EVT cells. Results obtained from *ITGB1* (encodes integrin-β1) knockdown and MMP-9 inhibition assays further indicated that MMP-9 and integrin-β1 might be the targets of BPA-induced migration. Similar inducible effect of BPA was also observed in a normal colon epithelial cell line [42].

Since, ERK and Akt are downstream effectors of integrins [43], we evaluated the effect of BPA on the activation of the MAPK and PI3K/Akt signaling pathways. Phosphorylation of ERK1/2 and Akt increased in HTR-8/SVneo cells after BPA exposure. The inhibitors of the MAPK and PI3K signaling pathways blocked BPA-stimulated cell migration and protein levels of integrin-β1 and MMP-9. Our results suggest that MAPK and PI3K signaling pathways might be involved in BPA-mediated stimulation of EVT cell migration *in vitro*.

BPA exerts detrimental effects on placentation in mice and rats [20, 22, 44]. “Low doses” of BPA (below 50 mg/kg body weight/day), defined by the National Institute for Environmental Health Sciences [45], were used in the present study. We showed that low-dose BPA increased integrin-β1 and integrin-α5 protein levels in the labyrinthine and decidua basalis layers. BPA exposure reduced the thickness of the labyrinthine and the spongiotrophoblastic layers, and increased the number of vacuoles above the spongiotrophoblasts. This was similar to Tait *et* *al*.'s findings that orally administrated BPA (50 mg/kg, from E1 to E11) led to increased vacuolization and reduction of spongiotrophoblast layer on E12 in CD-1 mice [23].

Blood vessels come to occupy the villous space in mice with the development of the villi [46]. The intervillous spaces within the labyrinth normally diminish in size as gestation proceeds as a consequence of the increasing density of the trophoblast branches [47]. In gene mutant mice with reduced branching and extension of the trophoblast villi, placentas attempted to compensate for the reduced nutrient transport by increasing fetal capillary density [46]. Consistent with the results of Tachibana et al.'s study [22], we noticed that the intervillous space in the labyrinth layer narrowed in the presence of BPA. Cross *et al*. suggested that vascular development was not the driving force behind labyrinth development in mice, but was the initiating molecular event in chorionic trophoblasts elucidating the scenario of labyrinth development [48].

During later periods of gestation in mouse, glycogen trophoblast cells differentiate within the spongiotrophoblast layer and subsequently diffusely invade the uterine wall. Mouse trophoblast giant cells (TGCs) are analogous to human EVTs, which ultimately differentiate into the polyploid giant cells of the placenta and exert their migratory and invasive properties [46]. We observed that the number of glycogenosomes within the TGCs was reduced in BPA-treated mice, which is similar with Tait *et al*.'s finding regarding depletion of glycogen storage [23].

In summary, we found that BPA promotes trophoblast migration and impairs placentation through a mechanism involving the up-regulation of integrin-β1 and MMP-9 and activation of the MAPK/ERK and PI3K/Akt signaling pathways *in vitro* and *in vivo*. Our results provide new evidence for the effects of BPA exposure on reproductive systems.

## MATERIALS AND METHODS

### Cell culture and drug administration

The EVT cell line HTR-8/SVneo was a kind gift from Dr. Charles Graham [29]. HTR-8/SVneo cells were maintained in Roswell Park Memorial Institute (RPMI) 1640 medium (Life Technologies, Carlsbad, CA, USA) containing 25 mM glucose, 2.05 mM l-glutamine, and antibiotics (100 U/ml penicillin, 100 μg/ml streptomycin) and was supplemented with 10% fetal bovine serum (FBS). HTR-8/SVneo cells were treated with BPA (Sigma, St. Louis, MO, USA) dissolved in dimethyl sulfoxide (DMSO; Sigma) for 48 h at concentrations of 10^–7^ M, 10^–6^ M, 10^–5^ M, and 5 × 10^–5^ M. DMSO was used as vehicle and its concentration in culture medium did not exceed 0.1%. After a 48-h BPA treatment, the cells were harvested or used for experiments described below. The specific MMP-9 inhibitor SC-311437 (Santa Cruz, CA, USA) was used to repress MMP-9. The selective MAP kinase kinase (MKK) inhibitor U0126 (Abcam, Cambridge, UK) and the PI3-kinase inhibitor LY294002 (Abcam) were applied to inhibit MAPK and PI3K signaling pathways, respectively.

### Animals and drug administration

Virgin Kunming mice (10- to 12-weeks-old) were obtained from the Laboratory Animal Centre, Chongqing Medical University (Chongqing, China). All experimental procedures were approved by the Ethics Committee of Chongqing Medical University. Mice were housed under a 12-h light and 12-h dark cycle with water and food ad libitum. Females and males were housed separately until mating. The first day of pregnancy (embryonic day E0.5) was defined as the day when a vaginal plug was detected. BPA (5, 10, 40 mg/kg/day) dissolved in corn oil (Sigma) was injected into the mice subcutaneously for 5 days from E0.5 to E5.5. Mice injected with vehicle from E0.5 to E5.5 were used as the controls. Placentas were excised under pentobarbital anesthesia on E14.5. Mean values of the thickness of each placenta layer were calculated using serial section from each of five individuals.

### Western blot analysis

Cells seeded in 24-well plates were lysed in 200 μl SDS lysis buffer (Beyotime, Shanghai, China). Lysed cells were denatured and boiled at 100°C for 10 minutes. Frozen placental samples were homogenised in radio immunoprecipitation assay (RIPA, Beyotime) lysis buffer supplemented with phenylmethanesulfonyl fluoride (PMSF, Beyotime). The cellular extract was incubated for 30 min on ice and then subjected to centrifugation at 12000 × g for 15 min at 4°C. The supernatant was collected, and the protein concentration was quantitated by BCA reagent kit (Beyotime) using bovine serum albumin (BSA) as a standard. Equal amounts of the protein samples (50 μg) were separated by electrophoresis on a 10% sodium dodecyl sulfate–polyacrylamide gel and transferred to a polyvinylidene fluoride (PVDF) membrane (Bio-Rad, CA, USA). After blocking with non-fat dry milk at room temperature for 2 h, the PVDF membrane was incubated overnight with antibodies (1:1000) at 4°C. Rabbit monoclonal anti-PCNA, anti-vimentin, anti-N-cadherin, anti-β-actin, and anti-phospho-Akt, Ser473 were purchased from Cell Signaling Technology (Devers, MA, USA). Rabbit monoclonal anti-phospho-ERK1/2, anti-phospho-Akt (Thr308), mouse monoclonal anti-ERK1/2, and anti-Akt were purchased from Millipore (Billerica, MA, USA). Rabbit monoclonal anti-integrin-β1 and anti-integrin-α5 were obtained from Abcam. Rabbit monoclonal anti-MMP-2 and anti-MMP-9 were ordered from Sanyin Technology (Wuhan, Hubei, China). After washing with Tris-Buffered Saline Tween (TBST)-20, the membrane was incubated with secondary goat anti–rabbit or goat anti–mouse IgG (1:1000, ZSGB-BIO, Beijing, China) at room temperature for 1 h. After three times of washing with TBST, the membrane was developed by enhanced chemiluminescence reagents (Millipore, MA, USA). Quantification was performed by densitometric analysis using Quantity One software (Bio-Rad).

### Proliferation assay

HTR-8/SVneo cells were seeded in triplicate at 1 × 10^3^ cells/well into 96-well microplates containing 100 μl RPMI with 10% FBS and exposed to different concentrations of BPA for 48 h. Cell proliferation was measured using a EdU imaging kit (RibBio, Guangdong, China). The number of EdU-positive cells was counted in five different fields (up, down, left, right, and middle) in each well.

### Flow cytometry analysis

Cell viability and cell cycle analyses were performed by flow cytometry using a BD FACS Vantage SE Cell Sorter (Becton-Dickinson, CA, USA), with a minimum 10,000 cells analyzed for each sample. The cell population of interest was gated on the basis of the forward- and side-scatter properties. Cut-off values (i.e., vertical and horizontal lines on the resulting scatter plots)were designated based on the autofluorescence of control cells. The data were analyzed using BD FACS Diva software (Becton-Dickinson). The results are expressed in terms of the percentage relative to controls.

### Scratch assay

HTR-8/SVneo cells were plated in 6-well plates containing 2 ml culture medium for 12–24 h. The confluent cells were used to generate a wound by scraping off the cells with a sterile pipet tip. The cells were then incubated in the absence or presence of different concentrations of BPA for 48 h and photographed after an additional 24 h and 48 h culture. DMSO (0.1% in medium) was used as a control for BPA treatments. At each of two time points, unattached cells were removed before photographing by removing the existing medium. The scratch width was calculated based on three replicates and analysis were carried out by blinded observers [30].

### Transwell cell migration assay

Cell migration assays were performed using uncoated Transwell insertsfitted with Corning membranes (8 μm pore size; Corning, Bedford, MA, USA) as described [31]. Briefly, HTR-8/SVneo cells were incubated in the absence or presence of different concentrations of BPA for 48 h. Then 1 × 10^4^ cells/300 μl of RPMI without FBS were plated in the upper chamber of each Transwell insert; the lower chambers contained 800 μl RPMI supplemented with 10% FBS. After 24 h culture, the cells in the upper chambers were removed by gentle swabbing. Cells attached to the membrane were fixed with ice-cold methanol for 30 min and stained with hematoxylin. The membrane was excised from each filter and mounted upside down on a clean glass slide. Cell migration index was determined by counting the number of stained cells in five randomly chosen fields from each membrane. Cell migration was tested in duplicate cultures and on three independent occasions.

### Villous explants and BPA administration

The use of the placenta tissues was approved by the Ethics Committee of Chongqing Medical University. Placental tissues at 6–8 weeks were obtained from women undergoing elective termination of pregnancy with gestational sac and original cardiovascular pulsation. Placental tissue was placed in ice-cold Dulbecco's Modified Eagle's Medium/F12 (DMEM/F12) medium (Life Technologies) with 100 μg/ml streptomycin and 100 U/ml penicillin and processed within 2 h of collection. The tissues were washed in sterile phosphate-buffered saline (PBS) and dissected to remove endometrial tissue and fetal membranes. Small fragments of placental villi were teased apart and placed on a transparent 12-well plate pre-coated with diluted Matrigel (1:19 in DMEM; Corning) and polymerized at 37°C for 30 min. Explants were cultured under 3% oxygen pressure and in DMEM supplemented with 100 μg/ml streptomycin, 100 U/ml penicillin and 20% FBS, with BPA (5 × 10^–5^ M) or DMSO (0.1%, as control group). Villous outgrowth was photographed every 24 h. The migration distance and the outgrowth area were calculated using Photoshop (Adobe, San Jose, CA, USA). Three separate experiments were performed in triplicate (N=9).

### Primary EVTs extraction and culturing

Primary EVT extraction was performed according to the protocol of Male et al. [32]. Briefly, the placenta villi were washed by PBS and digested with pre-warmed trypsin solution. Tissue suspension were placed on a 37°C-heat block and stirred with a magnetic stirrer for about 10 min. The digested tissues were filtered by 100 μm and 40 μm-bore diameter's filters (Corning). The filtrate was centrifuged for 5 min at 3000 rpm/min. Eight ml lymphoprep was added into the 15-ml centrifuge tube. The blown cell pellet was re-suspended in 5 ml Ham's F12 medium and gently added onto the prepared lymphoprep. The tube was centrifuged for 20 min at 4000 rpm/min. The cells were collected at the interface and washed with 15 ml Ham's F12 medium. The suspension was then centrifuged for 5 min at 3500 rpm/min. The cell pellet was re-suspended in 3 ml Ham's F12 medium to remove placental macrophages and incubated in 6-well plates for 20 min at 37°C. Finally, non-adherent cells were collected and seeded in the culture dish pre-coated with fibronectin (Corning) in medium containing 20% FBS.

### Real-time quantitative polymerase chain reaction (RT-qPCR)

Total cellular RNA was extracted using Trizol (TaKaRa, Dalian, China). A total of 3 μg of total RNA was subjected to reverse transcription by M-MLV reverse transcriptase (TaKaRa). The primers are shown in Table 1. RT-qPCR was performed using a SYBR Green Real-time PCR Master Mix kit (TaKaRa) under the following condition: initial pre-incubation at 95°C for 30 s, followed by 39 cycles at 95°C for 5 s and 60°C for 30 s. The relative mRNA levels of *ITGB1*, *CDH2*, *VIM* and *MMP-9* were analyzed using the 2^−ΔΔCt^ method.

**Table 1:** Primers used for RT-qPCR

Gene	Accession No.	Primer (5′–3′)	Amplicon length
*GAPDH*	NM_002046.5	Forward CAGGAGGCATTGCTGATGAT	138 bp
		Reverse GAAGGCTGGGGCTCATTT	
*ITGB1*	NM_033668	Forward CCTACTTCTGCACGATGTGATG	128 bp
		Reverse CCTTTGCTACGGTTGGTTACATT	
*CDH2*	NM_001792	Forward AGCCAACCTTAACTGAGGAGT	136 bp
		Reverse GGCAAGTTGATTGGAGGGATG	
*VIM*	NM_003380	Forward GACGCCATCAACACCGAGTT	238 bp
		Reverse CTTTGTCGTTGGTTAGCTGGT	
*MMP-9*	NM_004994	Forward TGTACCGCTATGGTTACACTCG	97 bp
		Reverse GGCAGGGACAGTTGCTTCT	

### Immunofluorescence staining

Cells were seeded on coverslips in 24-well plates containing 1 ml medium. Cells were fixed in cold methanol (100%) for 20 min, followed by blocking in 1% BSA in PBS for 60 min. Primary antibody (anti-N-cadherin, anti-vimentin, anti-integrin-β1, or anti-integrin-α5, 1:200) was incubated with cells in 1% BSA in PBS overnight at 4°C followed by addition of fluorescein isothiocyanate (FITC)-conjugated or Cy5-conjugated secondary antibodies (1:1000, Beyotime) for 60 min at room temperature. The cells were mounted in Vectashield medium containing DAPI (Beyotime). Images were obtained using a fluorescence microscope (Olympus, Tokyo, Japan).

### siRNA transfection

HTR-8/SVneo cells were transiently transfected with small interfering RNA (siRNA) sequences using HiPerFect reagent (Qiagen, Hilden, NRW, German) according to the manufacturer's protocol. The sequence of *ITGB1* siRNAs and scrambled control (GenePharma, Shanghai, China) was shown in Table 2. After 48 h of transfection, RT-qPCR and western blot assays were used to measure the knockdown efficiency.

**Table 2:** Sequences of siRNA used for knockdown assay

Gene		Sequence (5′–3′)
Scrambled control	Sense	UUCUCCGAACGUGUCACGUTT
	Antisense	ACGUGACACGUUCGGAGAATT
si-ITGB1 (human)	Sense	GGAGUUUGCUAAAUUUGAATT
	Antisense	UUCAAAUUUAGCAAACUCCTT

### Immunohistochemistry

Mouse placental tissues were fixed overnight in 4% paraformaldehyde (v/v). Fixed tissues were dehydrated in a graded series of ethanol, infiltrated with xylene and embedded in paraffin wax. Paraffin sections were prepared at the thickness of 5 μm and rehydrated in a graded alcohol series. Immunohistochemistry was carried out with the rabbit antibody kit (ZSGB-BIO). Briefly, antigen retrieval was achieved by placing the sections in sodium citrate buffer for 10 min at room temperature, followed by 15 min at 100°C in a microwave oven. Endogenous peroxidase was inhibited by incubation with 3% hydrogen peroxide for 10 min at room temperature. Sections were pre-incubated with 10% goat serum for 30 min at 37°C and then incubated with primary antibody (1:200) anti-integrin-β1 and anti-integrin-α5 at 4°C overnight. On the following day, sections were washed in PBS and incubated with biotinylated secondary antibody (ZSGB-BIO) for 30 min at 37°C and then with streptavidin-conjugated horseradish peroxidase (ZSGB-BIO) for 30 min at 37°C. Immunoreactivity was detected using the DAB substrate kit (Boster, Wuhan, China) and was visualized as brown staining. The sections were subsequently stained with hematoxylin (Nanjing JianCheng Bioengineering Institute, Nanjing, China).

### Statistical analysis

Results of densitometric analysis, cell counts and migration length are expressed as fold change or the percent variationcompared with the control. GraphPad Prism 5.0 was used for statistical analysis. Differences between groups were assessed by one-way ANOVA followed by Tukey's multiple comparisons test. All experiments were independently repeated at least three times. The results were considered significantly different at *P* < 0.05.

## SUPPLEMENTARY MATERIALS FIGURES AND TABLES



## Abbreviations

BPA: bisphenol A
EVT: extravillous trophoblast
MMP: matrix metalloproteinases
ECM: extracellular matrix
TGC: trophoblast giant cells
PCNA: proliferating cell nuclear antigen
MAPK: mitogen-activated protein kinase
PI3K: phosphatidylinositol-3-kinases
