# Supplementary material for: Bisphenol A exposure promotes HTR-8/SVneo cell migration and impairs mouse placentation involving upregulation of integrin-β1 and MMP-9 and stimulation of MAPK and PI3K signaling pathways
Source: Oncotarget. 2017 May 16;8(31):51507–21. doi: 10.18632/oncotarget.17882 (PMC5584264; doi:10.18632/oncotarget.17882)
Supplement: Supplementary file 1 [file oncotarget-08-51507-s001.pdf]

Bisphenol A exposure promotes HTR-8/SVneo cell migration and impairs mouse placentation involving upregulation of integrin-β1 and MMP-9 and stimulation of MAPK and PI3K signaling pathways

SUPPLEMENTARY MATERIALS

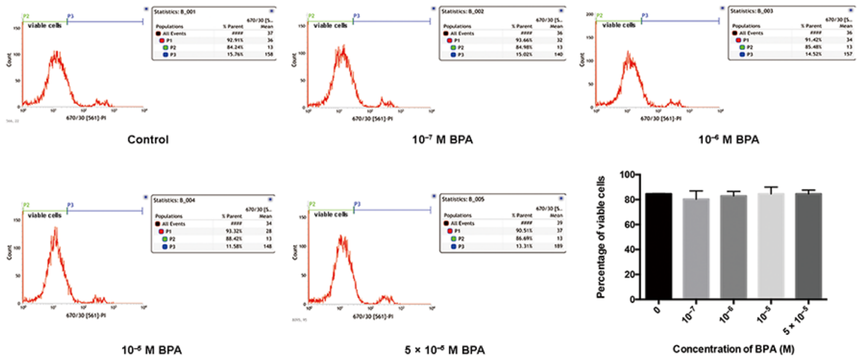

Supplementary Figure 1: Cell viability of BPA-treated HTR-8/SVneo cells as analyzed by flow cytometry. Cells were incubated for 48 h with BPA. The P2 values indicates the viable cells.

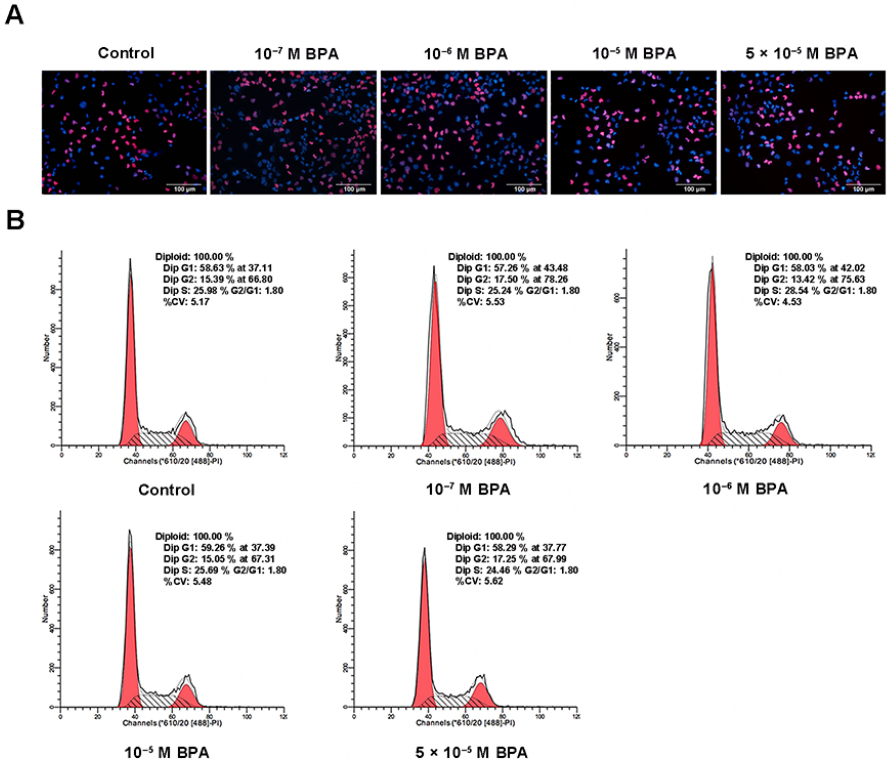

Supplementary Figure 2: HTR-8/SVneo cell proliferation following BPA treatment. (A) EdU assay detection of proliferative cells. Red indicates proliferating cells, labeled with fluorochrome Apollo, and blue (Hoechst staining) indicates cell nuclei. Scale bar, 100 μm. **(B)** Flow cytometry analysis of cell cycle.

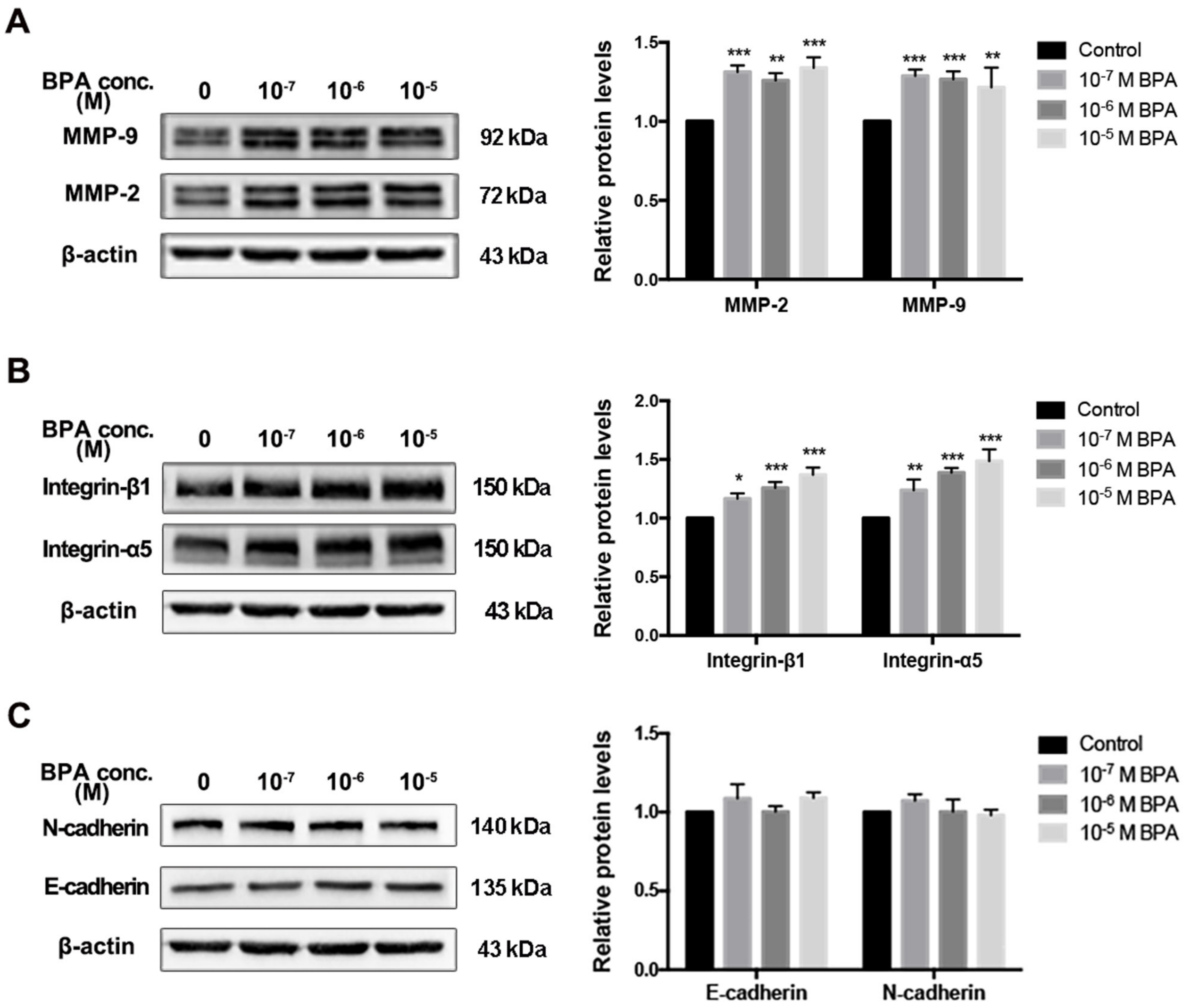

Supplementary Figure 3: BPA-upregulated protein levels in primary EVTs. (A) Western blot detection of MMP-9 and MMP-2 protein levels in primary EVTs. $^{**}P < 0.01$, $^{***}P < 0.001$. **(B)** Protein levels of integrin -β1 and integrin-α5 in primary EVTs. $^{*}P < 0.05$, $^{**}P < 0.01$, $^{***}P < 0.001$. **(C)** Protein levels of N-cadherin and E-cadherin in primary EVTs.

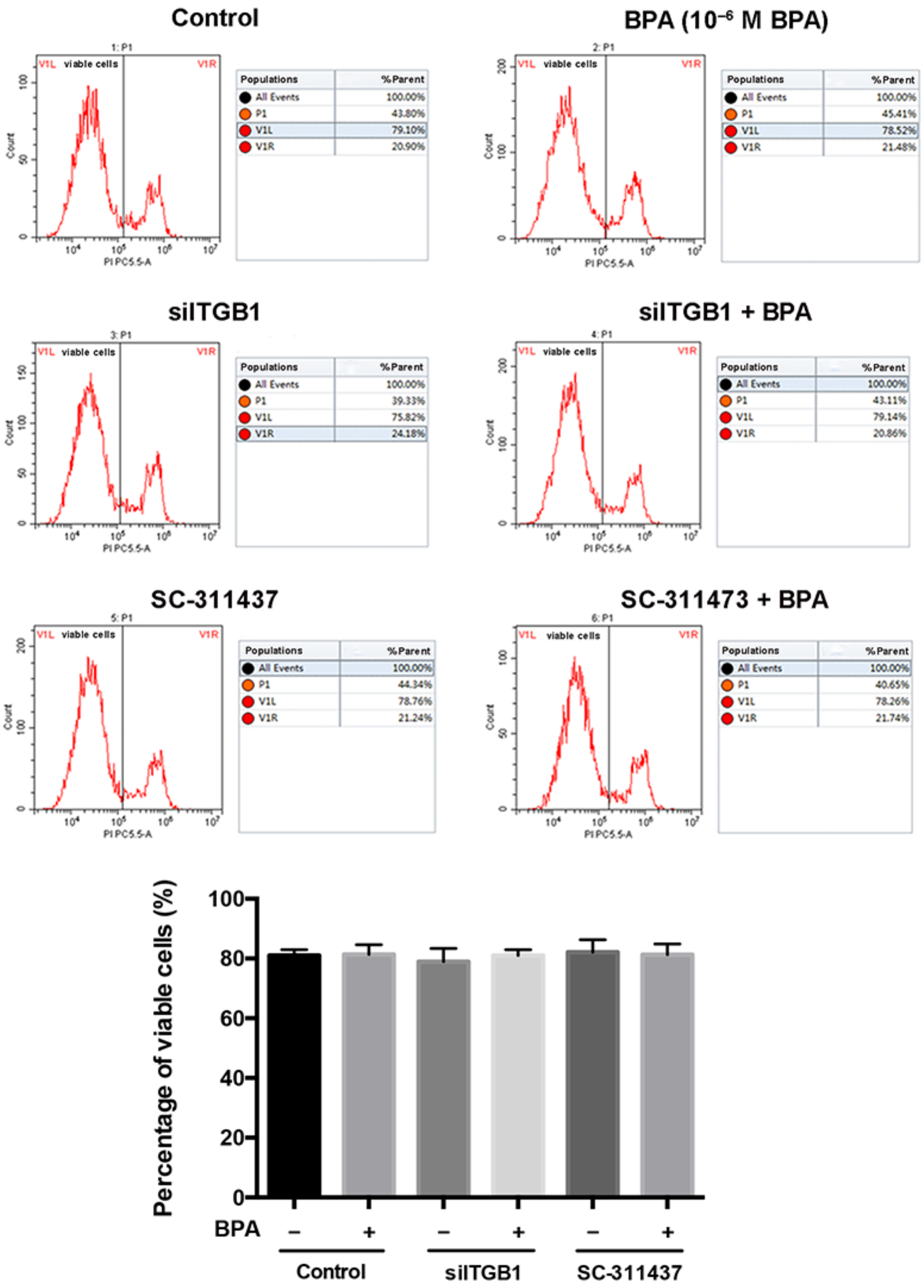

Supplementary Figure 4: Cell viability in HTR-8/SVneo cells treated with SC-311437 and si-ITGB1. V1L value indicates the viable cells. Cells were treated with siITGB1 (2 μM) and BPA (10^{-6} M) for 48 h, and with SC-311437 (5 μM) for 24 h before flow cytometry detection.

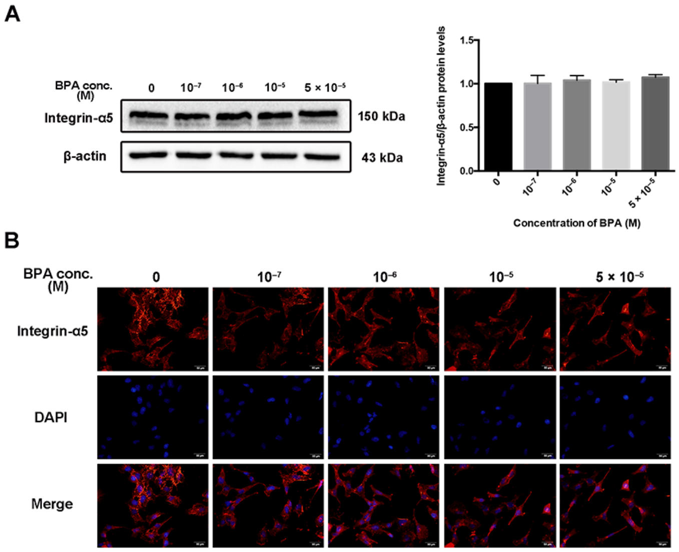

Supplementary Figure 5: Protein level of integrin-α5 in HTR-8/SVneo cells treated with BPA. (A) Western blot detection of protein level of integrin-α5 in HTR-8/SVneo cells treated with various concentrations of BPA. **(B)** Immunofluorescent staining of integrin-α5 in HTR-8/SVneo cells treated with BPA. Blue indicates DAPI staining, whereas red (Cy5) indicates integrin-α5. Scale bar is 50 µm.
